# The history of seed banking and the hazards of backup

**DOI:** 10.1177/03063127221106728

**Published:** 2022-06-29

**Authors:** Helen Anne Curry

**Affiliations:** University of Cambridge, Cambridge, UK

**Keywords:** seeds, data, seed bank, gene bank, backup, duplication

## Abstract

Seeds and other plant materials in seed and gene bank collections are rarely considered adequately conserved today unless genetically identical duplicate samples have been created and safely stored elsewhere. This paper explores the history of seed banking to understand how, why and with what consequences copying collections came to occupy this central place. It highlights a shift in the guiding metaphor for long-term preservation of seed collections, from banking to backup. To understand the causes and consequences of this shift in metaphor, the paper traces the intertwined histories of the central long-term seed storage facility of the United States (opened in 1958) and the international seed conservation system into which that facility was integrated in the 1970s. This account reveals how changing conceptions of security, linked to changing economic, political and technological circumstances, transformed both the guiding metaphors and the practices of seed conservation in these institutions. Early instantiations of long-term cold storage facilities vested security in robust infrastructures and the capacities of professional staff; between the 1960s and 1990s, this configuration gave way to one in which security was situated in copies rather than capacities. This observation ultimately raises questions about the security promised and achieved through present-day infrastructures for crop genetic resources conservation.

In the 1980s, the Nordic Gene Bank for Agricultural and Horticultural Plants established a storage facility for seeds in a coal mine on the Arctic island Svalbard. This was conceived of as a safe site in which to house a duplicate copy of the Nordic Gene Bank’s ‘base collection’ – its collection of seed samples (referred to as accessions) slated for long-term conservation – already being maintained in Alnarp, Sweden. The Alnarp base collection, in turn, consisted of genetically identical duplicate samples from several ‘active collections’ in regular use by crop breeders and other scientists from the Nordic region and around the world (Kjellqvist and Blixt, 1991; Yndgaard and Solberg, 2019). A proposal to create an Arctic seed storage facility for international use followed soon after. In gestation throughout the 1990s, this second facility, eventually named the Svalbard Global Seed Vault, took shape as an additional security measure for the conservation of diverse crop varieties held as samples in seed and gene banks worldwide. These institutions would be encouraged to create a copy of their collection for safekeeping in the vault (Fowler, 2016; Qvenild, 2008; see also Chacko, 2018).

Billed today as ‘the final back up’ (Crop Trust, 2020), the Svalbard Global Seed Vault represents the endpoint – for now, at least – of a technical recommendation of ‘safety duplication’ pursued by the crop genetic conservation community since the 1970s. Many seed banks that have contributed to the Svalbard Global Seed Vault collection were, at the time of its opening in 2008, already duplicating their collections for security, typically by dividing individual accessions to the collection into genetically identical subsamples and arranging for separate storage of the two. For several of the most prominent national and international banks, this duplication process included producing a local copy (often called a base collection) and also a complete duplicate collection held at a physically distant seed storage facility to comply with requirements for safety duplication (Peres, 2019). In such cases, a copy sent to Svalbard would not just be a backup copy, but a *duplicate* backup (safety duplicate) of a backup (the base collection) meant to secure a collection in routine use (the active collection).

Accounts of the Svalbard Global Seed Vault typically zero in on coldness, remoteness, and political stability as the salient features through which to understand the vision of security it offers. For example, Radin and Kowal (2017) see it as an archetypical example of ‘cold optimism’, an instantiation of ‘the belief that death – or the acceptance of its finality – can be postponed indefinitely through practices of [low temperature] preservation’ (pp. 9, 74). Together with other scholars, and consonant with the larger literature on biobanking, they pinpoint the alteration of seeds’ temporalities – the timeframes in which they are expected to live and evolve – as the key means by which security is achieved (Breithoff and Harrison, 2020; Harrison, 2017; Wolff, 2021; on other biological specimen collections, see, e.g. Radin, 2017; Van Allen, 2020). Complementary accounts note that the vault’s perceived security is amplified by its physical remoteness. LaFauci (2018) catalogs elements of secure isolation that pervade descriptions of the vault: It is located as far north as scheduled flights go, dug 100 meters into a mountain, and operates largely without onsite staff (pp. 25–26; see also Chacko, 2018; Hennig, 2019). The resilience conveyed in these depictions is accentuated by the idea that Norway is secure from the political disruptions that destabilize other countries. Breen (2015) points to ‘Norway’s history of political non-alignment, economic stability, and environmental preservation’ as significant contributors to the vault’s success with participating countries and institutions (p. 43; see also LaFauci, 2018).

It’s clear that coldness, remoteness and Nordic-ness are essential to the security promised by the Svalbard Global Seed Vault, and that each offers something different with respect to keeping open future options. These aspects are nonetheless distinct and, I argue, secondary, to the feature of the vault that is its main claim to effective conservation: duplication. Coldness and remoteness would provide little reassurance for unique (i.e. unduplicated) seed samples. Even when frozen, seeds will inevitably decay and die in storage. Without a field (in the right environment) and farmer (with the right knowledge) to regrow the seeds, both extremely improbable on Svalbard, passive freezer storage is security with an extended but still finite horizon. For the vault to realize security, its passive possession must be complemented by active maintenance, use and circulation of seeds. It can only ever be the final *backup*, a site from which to restore copies rather than warehouse originals.

Unlike coldness and remoteness, safety duplication is consistent across the operation of the global infrastructure for conserving crop genetic diversity. Not all crop genetic collections can have their natural lifespans extended through freezing – though scientists are trying very hard to make this the case (see, e.g. Walters et al., 2013). Not all seed banks are remote. By comparison, seeds and other genetic samples are rarely, if ever, considered adequately conserved today unless they have been copied. In what follows, I explore the history of seed banking in order to understand how, why and with what consequences the creation of copies came to occupy this central place. Undertaking this work raises new questions with respect to the nature of the security promised at Svalbard and across today’s international infrastructure for conserving crop diversity.

My account contributes to the rich literature, discussed above, on how the long-term security of seeds in cold storage facilities is established and projected. It does so by embedding an analysis of today’s seed banking practices within the longer history of state- and scientist-led efforts to conserve crop diversity. To date, historians’ accounts of efforts to conserve plant genetic materials have focused on the political and economic circumstances that made diverse crop varieties and collections of these valuable to scientists and states (Curry, 2022; Farnham, 2007; Flitner, 2003; Fullilove, 2017; Pistorius and van Wijk, 1999). In grappling with changing strategies in conservation, several scholars have noted the importance of guiding metaphors for crop diversity in determining the institutions created for and approaches taken: from ‘genetic resources’ to be mined to ‘ecosystems services’ to be maintained (Bonneuil, 2019; Fenzi and Bonneuil, 2016) to a ‘commons’ to be governed in the global interest (Saraiva, 2013). However, there is a further opportunity to understand how metaphors have shaped the practices of conservation *within* institutions and programs as well as the possibilities for their creation. Following recent ethnographic accounts that attend closely to the factors that shape conservation practices within contemporary seed banks (Chacko, 2019; Lewis-Jones, 2019), I look inside these institutions. I show that changing conceptions of security, linked to changing economic, political and technological circumstances, transformed both the guiding metaphors and the practices of seed conservation in these institutions. Early instantiations of long-term cold storage facilities vested security in robust infrastructures and the capacities of professional staff; between the 1960s and 1990s, this configuration gave way to one in which security was situated in copies rather than capacities.

To illustrate this transition, I tell the intertwined histories of the central long-term seed storage facility of the United States and the international seed conservation system into which that facility was later integrated. Both were key to the development of the dominant conservation strategies and institutions we rely on today – including the much-vaunted vault in Svalbard.

I begin by discussing the US National Seed Storage Laboratory, the first seed storage facility dedicated solely to the task of keeping seeds alive and available in perpetuity, to introduce the historical transition at the heart of my analysis: the shift from banking to backup as a conservation strategy.^1^ To explain this transition, I turn to the rise of duplication as a strategy for seed security within a nascent international system for conserving crop diversity. This rise tracked two increasingly prevalent Cold War survival strategies: first, dispersing and securing duplicate paper copies to preserve information and, second, installing duplicate infrastructural capacity as a communications and computing failsafe. As I suggest, the ascent of safety duplication in seed banks cannot be understood apart from anxieties about social, political and cultural survival during the Cold War and amidst catastrophic environmental change (Sepkoski, 2020).

Cold war catastrophism does not explain all, however. The 1980s saw seed banking crises, when a surfeit of samples, shortfalls in funding and strident critiques of the international conservation system threatened the work of long-term seed storage facilities. I show that backup served as an apparently cheap security measure through which these facilities and their conservation efforts could weather the storms occasioned by reorganization. Bringing these stories together offers an opportunity to reflect more generally, as I do in the concluding section, on the histories of both seed banking and backup, and also on the hazards of safety duplication – that is, of establishing conditions for security that valorize copying as a solution instead of addressing the root causes of insecurity.

## The Fort Knox of seeds

**Figure 1.**
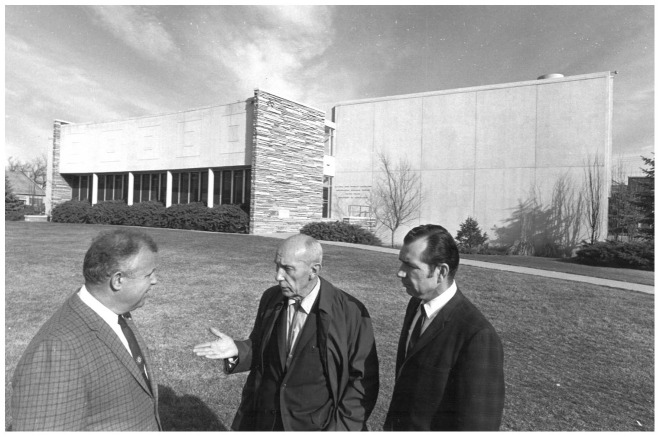
The National Seed Storage Laboratory around the time of its opening in 1958: Just another nondescript university building. National Laboratory for Genetic Resources Preservation Collection, Fort Collins.

In December 1958, the Colorado State University *News* announced the opening of a new facility, which it dubbed ‘The Fort Knox of the seed world’ (Anonymous, 1958). The recently completed National Seed Storage Laboratory (Figure 1), a US Department of Agriculture facility, had been tasked with making sure that valuable seeds would remain available to scientists in perpetuity. Hence, the reference to Fort Knox, otherwise known as the United States Bullion Depository, which took its nickname from the adjacent army post that secured it. Fort Collins was a city, not an active military base like Fort Knox, but the seed storage facility was, as I describe below, built like a bunker. Just as the substantial gold reserves at Fort Knox were essential to the country’s economic well-being, so too would the seeds stored in the Fort Collins laboratory be protected to sustain agricultural productivity. The suggestion of military defense was not the most salient aspect of the comparison to Fort Knox, however. Financial metaphors had run rampant in the years of lobbying that preceded the 1956 Congressional appropriation for the National Seed Storage Laboratory; I argue here that these metaphors crucially shaped its operations.^2^

Advocates for the facility maintained that the existing crop seed collections run by government employees and used to supply plant breeders with germplasm (that is, material to use in breeding programs) were like ‘the cashiers’ windows of banks, where funds are received and paid out’ – only collection managers were seriously hampered compared with typical cashiers. Without the capacity for long-term storage, these managers had to continually decide what to save and what to discard, and typically did so with only short-term interests in mind. ‘In short, they must operate a bank without a vault for reserve funds’, insisted advocates of a national seed storage facility.^3^ The laboratory at Fort Collins would be that vault. The seeds within it would be the reserve supply on which smaller local collections and the breeders who used them, and ultimately the US farmers who needed ever-better crop varieties, could always depend.

Metaphors were soon swept aside. ‘It really is a bank’, insisted an agronomist tasked with explaining to Congress the need for a seed storage facility. ‘[W]e want to be in a position where we can go to the bank and we can borrow some germ plasm’.^4^ Although officially labeled a seed storage laboratory, the Fort Collins facility would more often be described as a seed bank (or else a germplasm bank or gene bank), a label whose origins in metaphor are all but invisible today.

Agricultural administrators and scientists had been agitating for better seed storage facilities since at least the early 1940s. Some were motivated by a concern that new crop varieties developed by professional breeders were displacing local types that had been grown by farmers for generations. They thought that the resulting loss in diversity of types could pose a problem for future plant breeders, who might benefit from having a wide selection of starting materials in addressing new agricultural needs (Bonneuil, 2019; Curry, 2022; Fenzi and Bonneuil, 2016; Saraiva, 2013). Concern about this agricultural transition – which they saw as both inevitable and desirable – was compounded by their observation of typical practices. The United States had sponsored many plant exploration missions, successfully introducing hundreds and thousands of types into the country, but most collected materials were discarded if no immediate uses for them were found. USDA officials characterized this as a shortsighted strategy with respect to meeting future plant breeding needs and a waste of taxpayer dollars. Although maintaining seed stocks for breeding was, in theory, the responsibility of every breeding program and every breeder, they repeatedly observed that the routine seed maintenance meant to be ‘everybody’s business’ was, in fact, no one’s.^5^

Cold War politics added further layers of concern. The Soviet Union and Eastern Bloc countries had long been rich sources of genetic novelty for American breeders. These were, a USDA official noted, ‘the original home of our apples, pears, plums, alfalfas and many good forage grasses and legumes’.^6^ Introductions of wheat varieties from Russia in the late nineteenth and early twentieth centuries had played a crucial role in boosting production in the western states (Olmstead and Rhode, 2008). American breeders, dependent on imported germplasm for nearly all crops, given the paucity of native crop species, had maintained a good exchange relationship with Soviet colleagues up until the early 1940s. After 1945, reciprocal exchange slowed. The same was true, apparently, for China, another important source of plant germplasm. This posed a problem. As the same USDA official recalled, ‘the United States was losing a major source of breeding stock for most of its major crops’.^7^

**Figure 2.**
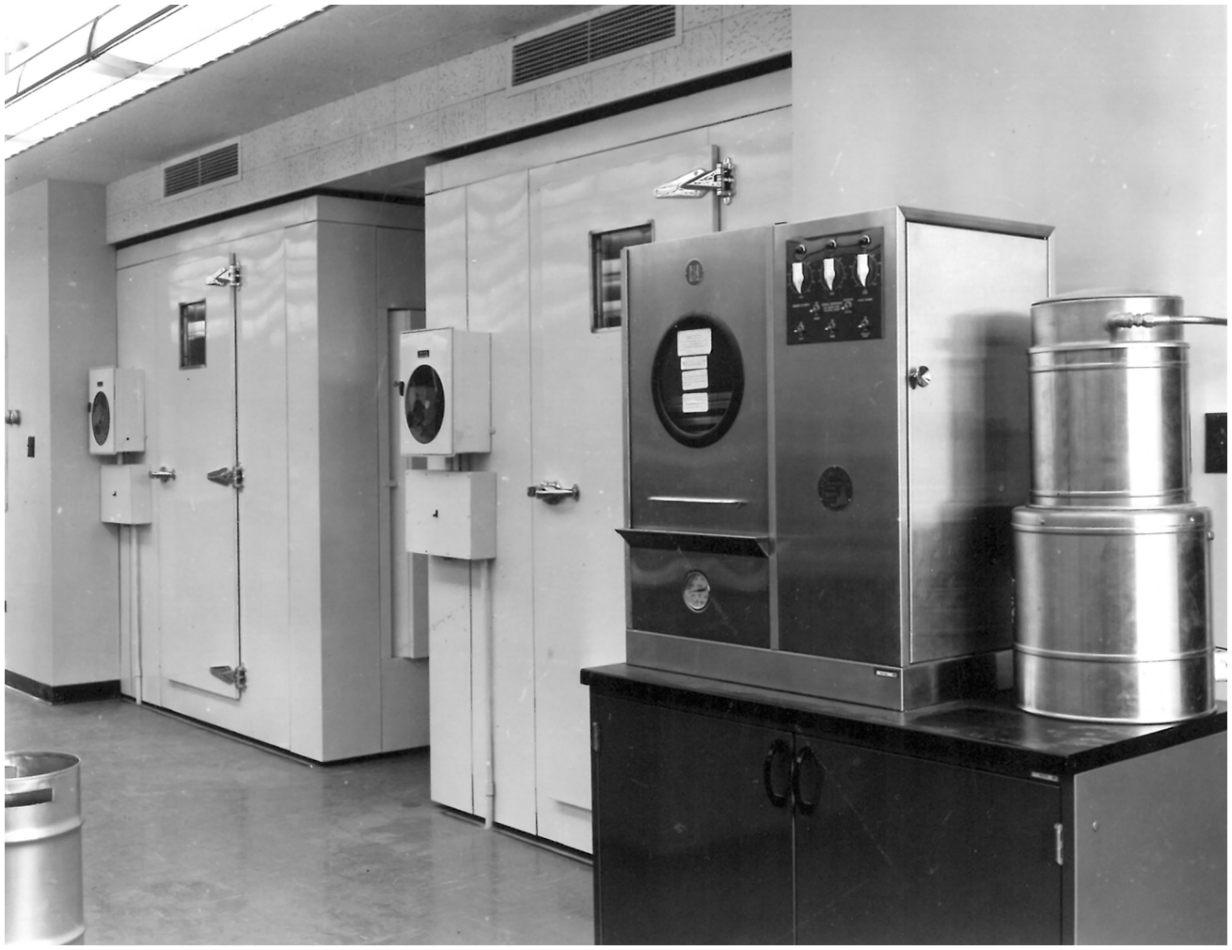
Entryways to the two cold storage rooms at the heart of the National Seed Storage Laboratory’s operations at the time of its opening. National Laboratory for Genetic Resources Preservation Collection, Fort Collins.

Advocates of the National Seed Storage Laboratory saw this institution as a way to stem the tide of losses associated with neglect and the hazards posed by the closing down of exchange. Old varieties could be kept extant even if no one was growing them. Breeders could hand over less-immediately useful materials from their collections, saving themselves the energy required to keep these viable, with the reassurance they would nonetheless be accessible in the future. ‘The Laboratory is not a warehouse or seed distributing center’, noted an early policy document for the facility, ‘rather, it is a germ-plasm bank for valuable stocks to be held over the years for the use of research workers when needed’.^8^ Its key assignment was simply to keep the seeds in its care alive for as long as possible. When the laboratory opened in 1958, this task was thought to involve organizing seeds in refrigerated storage (Figure 2), germinating them periodically to assess their continued viability and arranging for the provision of a fresh seed supply (typically from the original donor) when the viability of stored samples fell below acceptable thresholds. Since its mission revolved around the extension of seed viability, the laboratory also had a designated space for research on seed physiology and longevity. Here, two scientific staff members were expected to generate improvements in seed storage techniques (James, 1967a, 1967b, 1972).

Befitting its mandate to keep seeds safe and a Cold War context that fostered not only independence from foreign powers, but also safeguards against doomsday, the administrators who designed the National Seed Storage Laboratory buttressed it against external threats. The Rocky Mountain site was selected for climatic conditions – the naturally low humidity and temperature would reduce the costs of maintaining these artificially – and also martial strategy. The USDA’s only other national seed collection was in Beltsville, Maryland, close enough to Washington, DC to be considered vulnerable to military strikes.^9^ The vision of seed defense necessitated by potential warfare was actualized in walls of ‘heavily reinforced concrete’ that would ‘reputedly withstand an atomic blast at 3 miles’ (James, 1972: 398).

Reinforcement, in the sense of strengthening through the provision of supplemental resources or capacities, was integral to the facility constructed at Fort Collins. It had, as one technical report described, a standby compressor ready to be put into action should any of the refrigeration equipment break down and a diesel generator to ensure ‘current for essentials during prolonged power failure’ (James, 1967a: 102–103). In developing these safeguards, administrators hewed closely to security strategies adopted more widely in the Cold War-era United States. As Brett Spencer describes, planners who envisioned nuclear onslaught pursued a ‘triad of protective strategies’ to defend documents and artifacts they saw as essential to the continuation or re-creation of American life. These were vaulting, dispersal, and duplication – that is, the creation of bomb-proof storage spaces, the evacuation of materials to places unlikely to be targeted in an attack and the creation of copies (Spencer, 2014).

The only element of this triad not followed at the laboratory was duplication. On the contrary: Storing multiple samples of a single variety was considered wasted capacity.^10^ USDA administrators of the 1950s and 1960s were adamant that that the National Seed Storage Laboratory would not and did not duplicate existing storage capacities nor would it excessively duplicate seeds themselves. A USDA administrator insisted on this to a Congressional budgetary committee in 1955: ‘Therefore we repeat – the proposed National Seed Storage Facility will in no sense duplicate existing seed storage’.^11^ When a senator, wondering about the potentially escalating costs of this latter-day Noah’s Ark, questioned, ‘Are you going to get two of each one?’, he was re-assured that, no, the laboratory would hold ‘only one small package of each variety’.^12^ Ensuring resilience through redundancy applied only to physical infrastructure and equipment and, to a lesser extent, documentation and record keeping.

In sum, the National Seed Storage Laboratory, as initially imagined, was not a place for storing copies, but more like the bullion depository to which it was often compared: A secure repository for valued materials not in ordinary circulation. ‘Working collections’ – materials in active use by researchers – typically did not need to be sent to the laboratory as these were not felt to be in any danger from neglect.^13^ This remained the status quo for just over a decade.

## Networked security

In the 1970s, the USDA’s vision for how the National Seed Storage Laboratory would ensure the long-term availability of diverse plant genetic materials shifted. A handful of administrators and scientists now expressed concern that neither researchers nor their home institutions adequately prioritized preservation. In 1973, a subcommittee of the US Agricultural Research Policy Advisory Committee (ARPAC, an organization representing federal and state agricultural institutions) decried the lack of a federal policy on ‘genetic resources’, a failure which contributed to ‘most germplasm maintenance… being done in a haphazard, uncoordinated, and unsystematic way’ (Agricultural Research Policy Advisory Committee, 1973: 29). In the face of this general apathy, the National Seed Storage Laboratory could only achieve so much. One of the committee’s chief recommendations for ameliorating the situation represented a new perspective on duplication. It considered the deliberate copying of whole collections by dividing samples into genetically identical lots to be stored separately, perhaps even at distinct locations, ‘essential’ for long-term safekeeping. In other words, duplication was no longer unwanted redundancy but sought-after security. Within a decade, this new understanding had reshaped the purpose and practices of the US National Seed Storage Laboratory. The Fort Knox of seeds had begun its transition to a new set of guiding metaphors, no longer safeguarding a reserve supply, but instead providing redundant capacity, a feature increasingly referred to in communications and military circles as ‘back-up’. Here, I chart how and why this shift occurred, with particular attention to changing institutional and technological contexts.

Although it targeted US institutions, the 1973 report of the ARPAC subcommittee was a product of international concerns as much as national ones. For nearly 100 years, plant explorers and breeders had called attention to the possible loss of useful crop diversity, including locally adapted varieties maintained by farmers (sometimes called landraces) and crop wild relatives. They had also managed, in some cases, to institute large state collections for gathering and retaining access to this diversity (Bonneuil, 2019; Flitner, 2003; Lehmann, 1981; Saraiva, 2013). However, calls for international cooperation to preserve potentially disappearing crop varieties made little headway until the 1960s. It wasn’t until late in the decade that a number of factors converged to drive forward new urgent demands for collecting and conserving these plants, including concerns about population growth, environmental change and decolonization (Curry, 2022; Fenzi and Bonneuil, 2016; Pistorius, 1997). The single most important impetus, however, was the declaration of a worldwide revolution in agricultural production.

Most observers understood that increased crop yields seen in parts of Latin America, the Middle East and South Asia in the late 1960s – a so-called Green Revolution touted to be as socially and politically transformative as any red revolution – had been sustained, and would be extended, through the widespread cultivation of a handful of breeder-developed varieties (Curry, 2017b; Fenzi and Bonneuil, 2016). The sweep of breeders’ varieties across farmlands previously dominated by local lines, typically maintained by farmers from season to season, loomed with such certainty that scientists now referred to a process of ‘genetic erosion’. This would, experts insisted, irreversibly diminish the diversity of crop species and ultimately constrain the possibilities of future agricultural development. ‘What is inevitable and essential progress in one direction is a calamitous deprivation in another’, declared an influential gathering of agricultural and botanical experts in 1967 (FAO, 1969a: 13).

This statement reflected the collective assessment of the Panel of Experts on Plant Exploration and Introduction, a scientific advisory committee established in 1965 by the UN Food and Agriculture Organization (FAO). The initial impetus for assembling such a panel (first requested in 1961, authorized in 1965 and finally convened for the first time in 1966) had been to guide the FAO’s interventions in the international exchange of breeding materials. In 1967, when the Panel met in conjunction with a subcommittee of the environment-oriented International Biological Program, conservation of crop varieties loomed more urgently as an agenda item. This was largely thanks to the global agricultural transition its members believed was underway. However, as the involvement of the International Biological Program indicates, their concern was intensified by a more general and widely shared worry about the loss of biological resources on an ever more crowded and industrialized planet (Schleper, 2019). From 1967 onward, the Panel devoted its attention to developing and implementing an international plan for collecting and preserving crop diversity (Pistorius, 1997). Crucially, its aims were aligned with those of the Green Revolution, aspiring to keep breeders supplied with the materials needed for ‘modern’ varieties rather than to keep older ones in cultivation (Fenzi and Bonneuil, 2016).

The Panel’s core members, which included the US Department of Agriculture administrator in charge of US federal seed storage operations and others who joined them for the 1967 meeting, knew that the preceding decades had seen many seed collections established. They deemed these inadequate for long-term conservation because procedures for taking care of collections had rarely, if ever, been put in place. Otto Frankel, a wheat breeder and science administrator from Australia and chairman of the Panel, insisted that most collections of crop landraces and wild relatives were ‘distinctly less than effective’ at maintaining seeds and stocks in good condition and, as a result, ‘very few [could] be regarded as gene banks’ (Bennett, 1968: 9). Participants in the 1967 meeting regaled each other with stories of collections made and lost, such as the ‘shocking attrition’ over just a few years of a world collection of sorghum varieties, despite its being ‘in the hands of people who know their business’ (Bennett, 1968: 63; see also Harlan, 1972).

These experts’ shared distrust of typical collection management led the Panel to champion a new conservation infrastructure: A ‘coordinated international programme to conserve genetic resources’ could be created by linking existing ‘major seed storage installations possessing adequate technical facilities’ (FAO, 1969b: 4). The Panel agreed that breeders and other scientists with working collections should be encouraged to transfer or duplicate those collections to institutions with dedicated facilities and established procedures for long-term seed storage. The facilities deemed most secure would in turn be enrolled in an FAO-led scheme in which these more secure facilities agreed to store and possibly maintain seeds (that is, regenerate stored samples as these decayed or were depleted) that would serve as an internationally accessible long-term conservation collection (FAO, 1969b: 16–17). In the discussions that informed this plan, the US National Seed Storage Laboratory was repeatedly invoked as a model storage facility (Bennett, 1968: 74, 112, 178).

When the Panel set in motion a ‘Survey of Plant Genetic Resources in Seed and Plant Collections’, hoping to identify the existing materials and institutions that could be co-opted into its international effort, the results further cemented the view that existing collections were, by and large, failing at conservation. Although the survey revealed many more seed samples held in collections than anticipated, it also documented that ‘only a small proportion of maintenance centres possess (or use) special storage facilities (28.5 percent)’ and estimated that ‘350,000 samples are not stored under special conditions’ (Crop Ecology and Genetic Resources Unit, FAO, 1970a: 22). In other words, the effort to encompass as many collections as possible in the global survey generated new information about not only the location and extent of collections worldwide but also the varied – and usually suboptimal – conditions in which most were kept. These findings led to a renewed call for the movement of materials to sites deemed more secure: ‘It is very much hoped that the custodians of small collections … will take the initiative and, as soon as genetic conservation centres are available, send at least duplicates of their collections … for safe keeping under optimum conditions’ (Crop Ecology and Genetic Resources Unit, FAO, 1970b: 1).

The organizers of this survey surmised that its higher-than-expected tally of extant seed samples in collections reflected the fact that researchers around the world frequently exchanged seeds. Samples of wheat in a British collection were shared with German researchers, who would incorporate some into their own stores. Scientists developing maize in Mexico might acquire seeds from varieties found in Argentina or Cuba and, finding them valuable, decide to keep them on hand indefinitely. And so on. This implied that a system of deliberate, documented duplication would inevitably be accompanied by an existing and largely undocumented pattern of duplication. FAO coordinators were unconcerned about this eventuality. As they explained, ‘[al]though excessive duplication may have disadvantages, there are also advantages, in that samples’ duplication is the best kind of insurance against sample loss’ (Crop Ecology and Genetic Resources Unit, FAO, 1970a: 22).

Rather than resist duplication, the Panel of Experts on Plant Exploration and Introduction and associated FAO staff embraced it. In fact, as the Panel’s members labored to develop standards and procedures for conservation in the ensuing years, strategic copying of seeds became their formula for security. Scientists’ working collections, renamed active collections, were to be duplicated (typically by dividing accessions into two genetically identical samples) and the duplicates placed in long-term conservation collections, now distinguished as base collections. Seed kept in the latter would not ordinarily be used directly, for example, to distribute to researchers who requested it, but instead would supply the active collections from which routine multiplication and distribution of seeds would be made. Any research institution with a significant seed collection would be expected to have two copies of that collection, one of which served as the source of seeds used and circulated (the active collection) and a second, kept under best-possible storage conditions and accessed only rarely (the base collection) (FAO, 1973, 1975; see also Peres, 2019).

The duplication of all active collections in base collections would be accompanied by further duplication. Conserved material – that is, seeds in base collections – would *also* be duplicated, in a process eventually described as safety duplication. As a set of guidelines developed in 1975 set out, ‘Arrangements must be made for the duplication of conserved material in order to minimize the danger of loss’. There were two options. The duplicate set of seeds could be ‘accept[ed] for storage in the packaged form in which the material has been received’. This system, in which materials would never be touched unless requested or updated by the originating institution, was subsequently known as a black-box arrangement. A second option was for the duplicate set of seeds to be fully incorporated into the existing collection at the institution that received them (FAO, 1975: 31). Regardless of which option was selected, the expectation was that any institution with a base collection would make a complete copy of this collection and arrange for its storage elsewhere, a precaution that would mitigate against catastrophic events like floods, fires and wars, any of which might wipe out whole collections in a single stroke (FAO, 1975: 23).

The procedures of safety duplication depended on, and instantiated, close connections among geographically scattered and geopolitically divided facilities. In its earliest recommendations, the FAO Panel spoke of connecting new and existing institutions via a ‘coordinated international programme’ operating on a ‘mutual and cooperative basis’ with central oversight of seed conservation, maintenance and exchange from FAO (e.g. FAO, 1969b). By the early 1970s, its vision of coordinated cooperation and mutual aid was recharacterized as a ‘network’ (FAO, 1973) in which both plant materials and information would ‘flow’ among participating institutions (FAO, 1975: 33; Peres, 2019).

This network ideal merged an established (and complementary) concern at FAO about the importance of good documentation and information exchange with the Panel’s interest in a cooperative, multi-institution conservation program. Two FAO consultants had promoted the former at the 1967 meeting of the Panel, describing the new capacities of electronic computers and advocating the creation of an ‘international network of information centres’ that would ‘allow information about world plant genetic stocks to be relayed between any of the units with ease’ (Bennett, 1968: 49). Enthusiasm for the electronic transfer of seed- and plant-related data through a network of computing centers drew inspiration from contemporaneous experimentation with such networks among engineers, computer scientists and military planners (Abbate, 1999). And it took shape alongside efforts to integrate computing and networking technologies into many areas of biological research, including the management of biological specimens and data about these (García-Sancho, 2012; November, 2012; Strasser, 2019).

The subsequent adoption of the network concept to encompass seed storage and exchange (in addition to data exchange) not only aligned the imperatives of seed conservation and information management, but also signaled the integration of crop diversity conservation into the emergent network society of the late twentieth century (Castells, 2010). A critical influence in this direction came through the Consultative Group on International Agricultural Research, or CGIAR. Founded in 1971 and championed by the World Bank and major state and philanthropic donors as the international coordinating body for agricultural research and development aid, CGIAR adopted the language of networking from its first formal gathering.^14^ It established an International Board for Plant Genetic Resources (IBPGR) in 1974, tasking this body with promoting an ‘international network of genetic resources activities’ (IBPGR, 1975: 1).

IBPGR ultimately displaced FAO and its Panel of Experts on Plant Exploration and Introduction from their central positions in the realm of crop conservation (Curry, 2017b). The ascent of IBPGR did not, however, displace the vision of the Panel with respect to the centrality of duplication to effective conservation. The standards and guidelines it established for its emerging genetic resources network required collectors who received funding from IBPGR to leave duplicate samples in any country they visited. More importantly, it adopted the division of labor, made possible through duplication, of active and base collections, and encouraged safety duplication of entire base collections. In a further adaptation of the concept of a base collection, IBPGR invited several national and regional institutions to hold ‘world base collections’ – that is, to become the designated global repository of a particular crop or crops. For institutions who accepted, this implied holding an aggregate duplicate of all the world’s (or, as was more often the case, an entire region’s) active or working collections for a subset of crops. It did not mean that other institutions *not* designated as world base collections would relinquish their own holdings (IBPGR, 1977: 38).

Thanks to the wariness of funders about the costs of keeping these collections and the understandable sense of work proliferating, IBPGR remained vigilant (at least on paper) about other kinds of redundancy that might be implied with respect to its work. Its initial terms of reference from CGIAR insisted that it was to navigate within existing programs so as ‘to avoid unnecessary duplication’ (IBPGR, 1975: 15). However, this wariness applied chiefly to the organizational efforts of IBPGR as a whole and to the activities it set in motion at particular sites, and not to seeds and collections. In the 1970s, the only duplication of seed samples in collections that was seen as a problem was careless regeneration or multiplication of seed stocks. Growing out stored seeds either to replenish supply (regeneration) or to distribute them (multiplication) inevitably brought changes in the genetic constitution of the sample. The whole purpose of duplication in a base collection, kept in ideal conditions and minimally disturbed, was to limit the exposure of seeds to these other kinds of biological reproduction (Cromarty et al., 1990). Untouched copies of seed samples safely stored were the bedrock of the system’s promised security.

Today’s readers will recognize this security strategy as backup. Historians and media studies scholars trace the roots of twenty-first century digital backup culture to the Cold War. Fears of nuclear attack on key institutions created conditions conducive to the dispersal and vaulting of carbon copies, as described above (Spencer, 2014; see also Murphy, 2014). Infrastructural redundancy was also thought essential to a range of military operations. Missile defense demanded redundant generators and identical computers working in tandem, each replicating the capacity of the other (Edwards, 1996: 104–106). As NASA undertook manned space travel, its engineers provided substitutable versions of nearly every aspect of the mission, even the astronauts (Brennan, 2016). And – perhaps most salient for understanding the developing seed conservation network of IPBGR in the 1970s – when engineers began developing ‘survivable communications systems’ for use by the US military they looked to distributed systems in which the loss of a single node or even several would not disrupt communication (Abbate, 1999; see also Galison, 2001). This meant, among other features, ensuring that each node in the network was connected to multiple others. For example, a 1964 design for a system of highly connected nodes transmitting information through a mode later described as ‘packet switching’ – an operation adopted in the earliest internetworks – envisioned ‘primary’ links between nodes, as well as ‘back-up links’ and ‘second back-ups’ (Abbate, 1999: 12).

A genetic resources conservation network constituted in part through deliberate duplication reflected the increasing prominence of this strategic Cold War approach to security. Although they had not (yet) adopted the language themselves, the scientists planning and overseeing the creation of the IBPGR network of world base collections (later described as its global network of gene banks), were envisioning duplication in collections in much the same way as military engineers were envisioning the backup elements being built into contemporaneous defense and communications systems. As detailed in the official IBPGR handbook on the design of seed banks, first prepared in 1976, ‘duplicate collections (of base collections) for long-term conservation… are housed for security in different locations from corresponding base collections’. Although the handbook recommended that original base collections be sited in a ‘socially stable area within reach of security personnel’, on solid high ground unlikely to quake or flood, and at a distance from ‘dangerous chemical or fuel storage areas’, even careful location selection could not guarantee survival. Hence duplicate collections maintained elsewhere were needed as the ultimate ‘insurance against accidental loss’ (Cromarty et al., 1990: 1, 70, 91).

The US National Seed Storage Laboratory was among the first institutions to be identified by IBPGR as possessing the right mechanical and managerial infrastructures to house a world base collection (IBPGR, 1977: 38). The US laboratory’s intercalation with the international network hastened its adoption of the language of active and base collections promulgated by the FAO Panel of Experts on Plant Exploration and Introduction and IBPGR, along with the practices of strategic duplication and dispersal of samples that both espoused. Meanwhile, as the promise of networked security grew, the appeal of banked security plummeted. The 1970s saw rising interest rates, successive oil shocks in 1973 and 1979, deregulation of banks, and the first major bank failures since World War II (Grossman, 2010: ch 10). In the United States, Americans’ uncertainties about the reliability of the banking system were cemented in the 1980s as the so-called savings and loan crisis unfolded, with small banks closing by the hundreds (Grossman, 2010: 269–272). Even the iconic vault at Fort Knox now seemed irrelevant, with the United States abandoning the gold standard in 1971.

**Figure 3.**
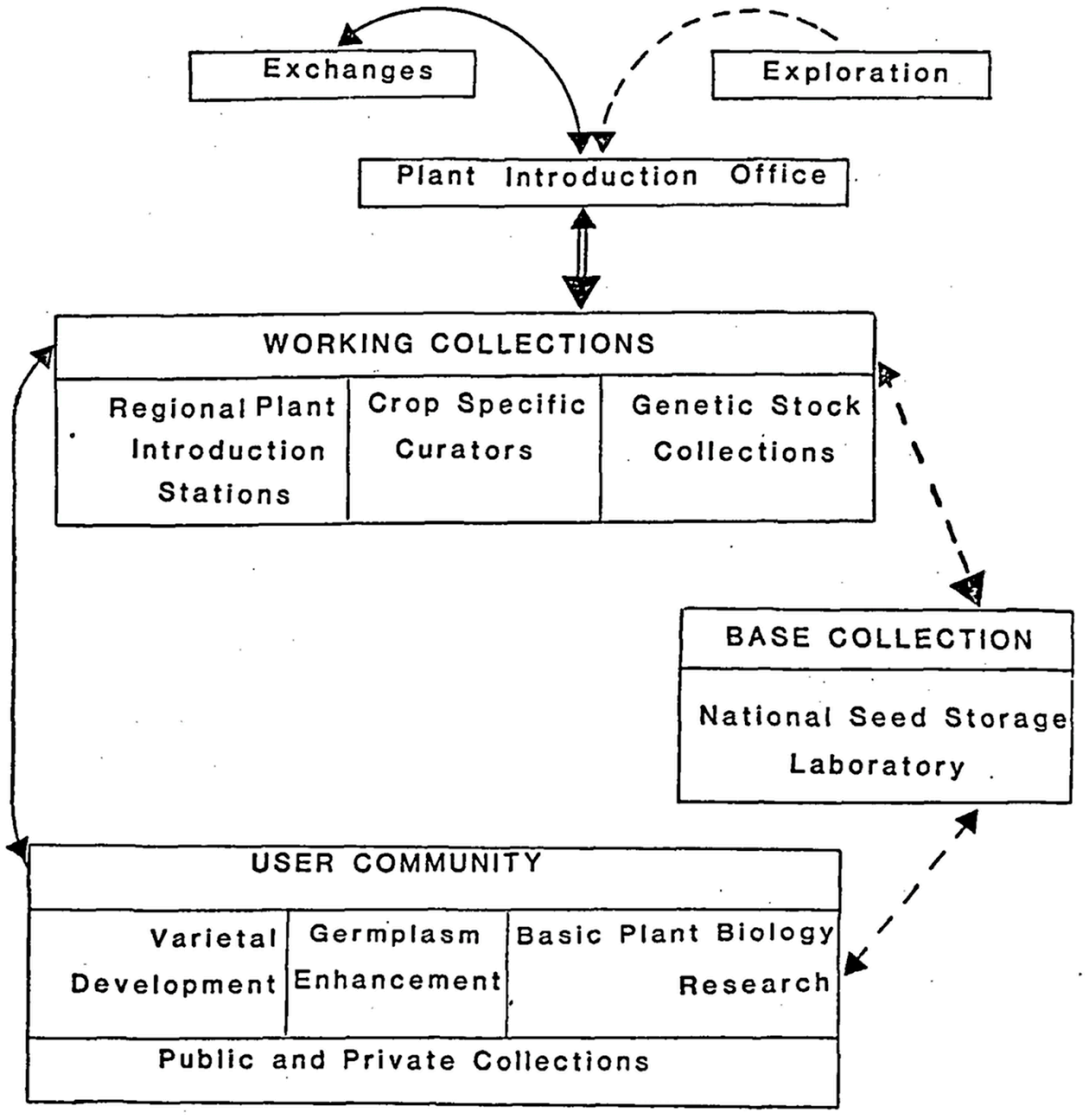
Known duplication defined the effective functioning of the US National Plant Germplasm System by 1983. Plant breeders and researchers (the ‘user community’) were supposed to obtain seeds from official ‘working collections’, which, in principle, would have all material introduced into the United States ready to share. However, just in case, the working collections were also backed up in the national ‘base collection’, the National Seed Storage Laboratory. From Wilkes and Williams (1983: 155), and used by permission of Taylor & Francis, Ltd., www.tandfonline.com.

Given this backdrop, it’s hardly surprising that in the same period, the metaphor used to characterize the activities of the National Seed Storage Laboratory shifted, from that of a bank’s reserve funds to that of a backup copy. In the mid-1970s, administrators and scientists in the United States Department of Agriculture reconceived the National Seed Storage Laboratory as a base collection (Figure 3), one that served a US network of institutions, researchers, plants and seeds newly dubbed the ‘National Plant Genetic Resources System’. A 1981 guide to the laboratory’s operations reminded would-be users: ‘Base collections are not intended to meet the day-to-day needs of plant breeders and other plant scientists, but rather are reserve stocks. … Generally, seed samples in base collections are also held in a working collection and only distributed when unavailable from some other source’. The overview also reminded citizens that US collections were in wide demand, with the result that ‘our NSSL [National Seed Storage Laboratory] serves as a primary base collection or as *a back-up* base collection (long-term maintenance) for crops that are important worldwide’ (USDA, 1981, emphasis added).

## The seed banking crisis

The unquestioned ascent of the Cold War security-through-duplication strategy proved short lived, undermined by postcolonial protests and especially the dwindling resources afforded to state infrastructure in an increasingly neoliberal age. Even as researchers and administrators keen on conservation celebrated the apparent successes of the new global network of world base collections (e.g. Hanson et al., 1984), and with it the duplication of seeds from places and institutions seen as insecure into seed storage facilities judged state of the art, this process of copying and storing to remote locations came under intense scrutiny. Duplication, recently established as the core of a successful international network of seed conservation, came to be seen as a liability. This led to circumstances in which experts recognized duplicate seed samples as both the foundation of successful conservation and a threat to the same.

The 1980s saw a powerful surge in critiques of, and resistance to, the world conservation network sought by IBPGR and its funders, which eventually forced its reimagining (Aoki, 2008; Curry, 2022; Fenzi and Bonneuil, 2016). In 1979, the Canadian activist Pat Mooney linked IBPGR sponsorship of collecting missions and the ferrying of seeds to well-resourced facilities in the US and Europe to a long history of imperial exploitation. ‘The emerging network of gene banks takes national genetic treasures from the Third World to be stored abroad’, he noted (Mooney, 1979: 30–31, 102). In other words, the world network of base collections was best understood as a means of securing pirated copies rather than safety duplicates. The accusation of biopiracy – the idea that foreign governments and, increasingly, private concerns were plundering the biological resources of economically and politically weaker states – resonated strongly in countries still reeling from the legacies of colonialism (e.g. Shiva, 1997). In this view, achieving security for seeds through duplication, as pursued by IBPGR, contributed to insecurity for many states and, crucially, farmers as well.

The fight over the control of seeds supported by activists like Mooney and pursued by the non-aligned states at FAO in the 1980s (and continuing thereafter) generated new audits of seed banks. Scientists and administrators associated with IBPGR needed to provide evidence that their work had been in the global interest and that its network of collections was indeed keeping seeds safe and accessible to all potential users. Critics, meanwhile, needed proof that such claims were pure fiction. Subsequent accounts compiled many shortcomings of national and international conservation efforts: broken refrigeration systems, lost samples, restrictions on access (e.g. Goodman, 1984; Mooney, 1983; US Comptroller General, 1981). Even champions of the existing structure had to acknowledge that the putative success of gathering seed samples had created a significant influx of materials to conserve, and that this multiplied the labor needed in processing, monitoring and evaluating samples, while funding for such work remained stagnant (e.g. Frankel, 1984; Peeters and Williams, 1984). What is more, the burgeoning size of collections had not been accompanied by increasing demand, often because samples lacked adequate documentation. A 1984 study of seed and gene bank use conducted by IBPGR described ‘a consensus of opinion that genebanks are not being used very extensively by breeders’ (Peeters and Williams, 1984: 22).

**Figure 4.**
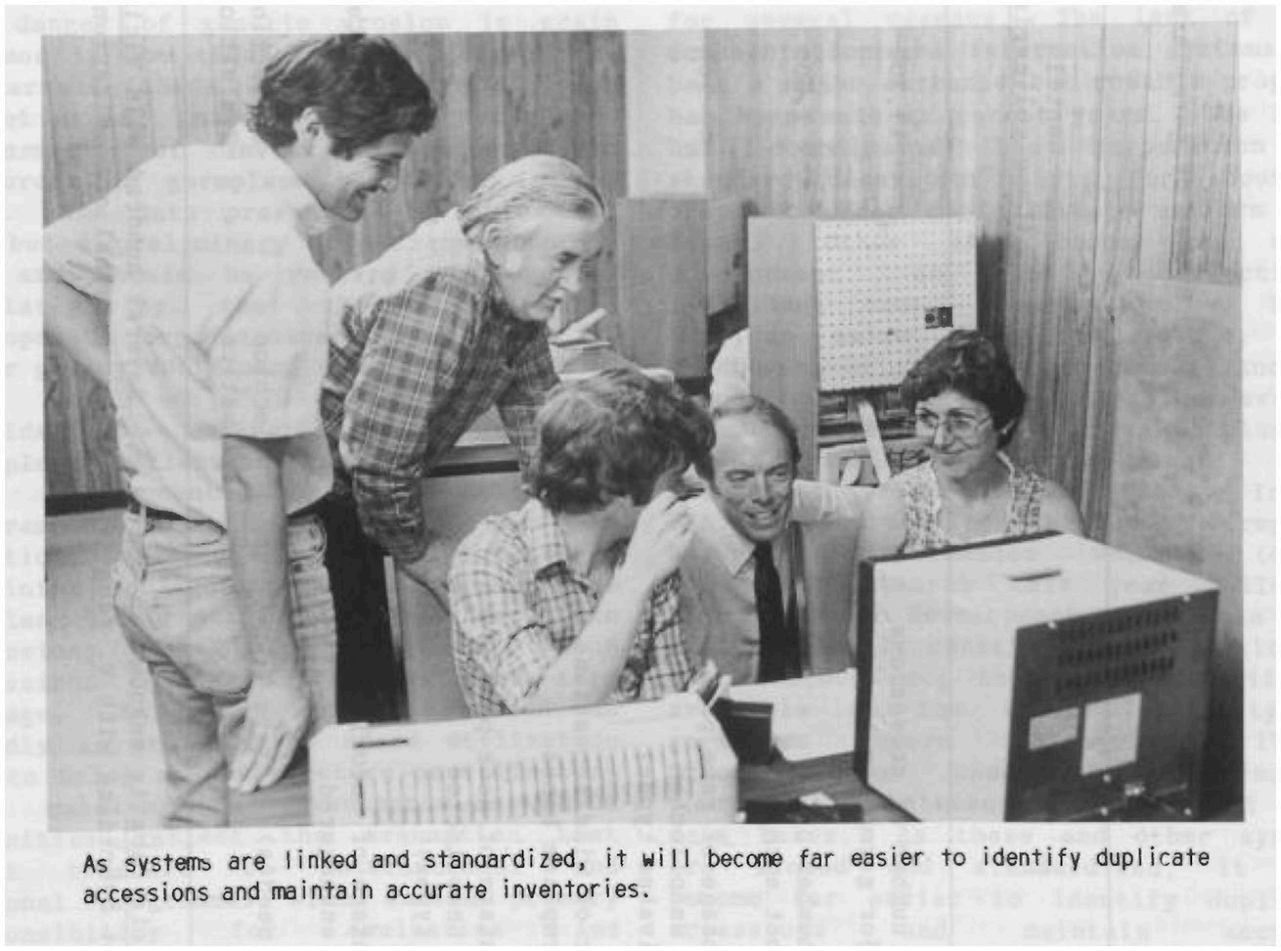
Networks, standards, computerization: All would help identify system-threatening duplicates. From *Plant Genetic Resources Newsletter* 60 (1984). Used by permission of Bioversity International.

A further study identified duplication as a chief culprit in seed banks’ failings. ‘At least 50 percent of the combined collections of most crop species are duplicate accessions’, the author calculated. In the case of ‘valuable collections’ this was good, as it ‘protected against loss from natural disasters, human error or political upheaval’. As a system-wide phenomenon, however, it presented a problem. ‘[I]ndiscriminate duplication of entire collections at numerous genebanks is costly and unnecessary’, the report suggested. ‘Redundant duplicates within the same bank are undesirable’ (Lyman, 1984: 5). This view pointed toward a procedure that an increasing number of crop conservation specialists espoused: rationalization of collections. Rationalization would involve, among other means of streamlining collections, the elimination or reduction of duplicate samples (see Figure 4). Unfortunately, eliminating duplicates also necessitated reliable information on the content of collections, and consistent, comparable, reliable data were all but impossible to obtain (Peeters and Williams, 1984).

The acknowledgment that seed banks were both struggling to stay abreast of maintenance and falling short in their expected provision of services to plant breeders invited proposals for reimagining germplasm conservation. In a clear signal of the changing tides, Otto Frankel, the early and effective champion of a crash program of global collection through his chairmanship of the FAO Panel of Experts on Plant Exploration and Introduction, proposed a change in direction. The use of collections, he thought, was especially hampered by the lack of information about them. At the time, IBPGR estimated that 95 percent of samples in gene banks had no agronomic evaluation data attached (Peeters and Williams, 1984: 24). However, it was absurd to expect that seed banks, with their limited resources, would be able to produce such data for the thousands of samples they now maintained. The known circumstances of unwanted or undetected duplication only enhanced the untenability of this approach: Scientists imagined many cash-strapped institutions repeating the same laborious characterization process for identical samples. Frankel’s proposed solution was instead the ‘rationalization of evaluation’ through the selection of a ‘core collection’ of samples that were thought to represent most of the genetic diversity in the collection. This would entail ‘a drastic reduction in redundancy’, at least in terms of genetic variation within the identified core collection and reduce the resources required for evaluation (Frankel, 1984: 161; see also Brown, 1989, 1995).

The core collection concept found influential champions in the 1980s and 1990s (Brown and Spillane, 1999). A chief selling point was not that it would reduce the overall number of samples, but that it would ensure that the widest possible range of genetic diversity would be maintained and used, even in circumstances of constrained resources. In fact, the core collection concept appealed precisely because it meant that streamlined gene bank management could occur without a costly investment in eliminating duplicate samples via biochemical and molecular techniques. In the early 1990s, these were, for the most part, prohibitively expensive to run for entire seed bank collections (National Research Council, 1993: 172). In other words, core collections would allow duplicates to persist without undermining effective conservation.

In the absence of quick and effective ways of identifying duplicate samples, gene bank managers mostly had to live with them and attempt to forestall further duplication by remaining vigilant at the moment of accession to a collection. Meanwhile, many within the international plant genetic resources community felt that the answer to the challenges of effective seed banking lie less in pushing managers toward rationalization than in providing adequate resources. Meeting the costs of keeping existing accessions extant, even planning to do so in perpetuity, was not beyond the realm of possibility, and for many crops the marginal cost of each additional accession in a major gene bank collection was low (see later estimates in Koo et al., 2002, 2003).

Nonetheless, in an institutional environment shaped by demands for efficiency and utility, the ominous cloud of unwanted and unidentifiable duplicates would not dissipate. The FAO’s first major report on the State of the World’s Plant Genetic Resources for Food and Agriculture noted that estimates of duplication across global collections ran to more than 35 percent. Although keeping known copies, in the form of safety duplication, was desirable, the report reminded readers that ‘unintentional or overduplication is wasteful and should be minimized’ (FAO, 1997: 111). The second such report, published more than a decade later, announced that much of the increase in gene bank accessions worldwide was the product of ‘exchange and unplanned duplication’; it estimated the number of *distinct* accessions (rather than the duplicates) at ‘less than 30 percent’ of the 7.4 million on the books (FAO, 2010: xix). Indeed, growth was good, but the ‘backlog in regeneration and over-duplication continue[d] to be areas of concern’ (FAO, 2010: 3).

In a novel twist, the 2010 FAO report also identified excessive *safety* duplication as a potential issue, even as it celebrated the successes of safety duplication in shoring up global conservation as a whole. Thanks to the unusually comprehensive work of a group of barley researchers, the extent of duplicate safety duplicates was well established for this crop (Curry, forthcoming). As the FAO (2010: 74–75) report described:

Considerable safety duplication exists among the four largest barley collections; those of PGRC [Plant Gene Resources of Canada], USDA, Embrapa [Brazilian Agricultural Research Corporation] and ICARDA [International Center for Agricultural Research in the Dry Areas]. There is a large overlap between the Canadian and USDA collections following safety duplication of the USDA collection of oats and barley in Canada in 1989 and the Brazilian collection is mostly integrated into that of the USDA. The ICARDA collection is to be duplicated in the SGSV [Svalbard Global Seed Vault] as a second level of safety. … 33 percent of this collection is already duplicated at CIMMYT [International Center for the Improvement of Maize and Wheat] and 65 percent is duplicated elsewhere. … The duplication of accessions among collections, whether planned or unplanned, may result in large numbers of common accessions among different genebanks which, in turn, may be duplicated again as part of the planned safety duplication of entire collections.

Ultimately, concerns about too much duplication cast safety duplication into shadow as well, and did so even as the Svalbard Global Seed Vault, opened for deposits in 2008, promised to take such duplication to the next level. Failure to provide seed and gene banks with the resources they needed to do their work well had generated circumstances in which backup was, paradoxically, both security and a liability.

## Conclusions

In the fifty years from the creation of the National Seed Storage Laboratory to the opening of the Svalbard Global Seed Vault, the dominant metaphor used to explain long-term storage and preservation of germplasm as seeds, in the United States and beyond, shifted. The once-dominant comparison to the cashier’s window of the local savings and loan bank – or indeed in the United States to the secure vaults of the Fort Knox gold reserve – lives on in the labels ‘seed bank’ and ‘seed vault’. However, the most common active metaphor for long-term seed storage is that of backup. The practices of conservation shifted as well. Seed storage facilities, and the people who operated them, were initially seen as the safeguards against loss. Robust, state-of-the art facilities and trained technical staff would oversee the never-ending maintenance regimes needed to secure collections from decay. When institutions were routinely left without the resources necessary for their work, security was increasingly vested in copies, rather than in institutional or professional capacities.

In the age of cloud computing, backup often looks both cheap and easy, a task that requires individual or institutional discipline rather than significant financial outlay or skill. As such, it seems like a suitable strategy for collections and curators. Seeds, like computer files, are vulnerable to loss, but, apparently, a breeze to copy. Why not copy, then? Well, for one, copies are not as cheap as often imagined. Copies of business and government records stored in remote vaults after World War II, chiefly so that the world could (it was thought) be reconstructed after a nuclear attack, often became out of date in storage, necessitating elaborate information management systems so that storage did not become its own form of annihilation (Spencer, 2014). The transition to digital has, if anything, intensified concerns about the need for careful management of copies slated for preservation (Bradley, 2007). Meanwhile, strategists assessing policies of deliberate institutional and infrastructural redundancy – typically instated as a means of enhancing security – in fact create new possibilities for accidents, not least because creating duplicate capacity makes a system appear more secure and therefore encourages greater risk taking (Sagan, 1995).

A growing literature on the environmental costs of computer backup raises further cause for concern. The data centers that constitute the ostensibly ethereal realm of cloud computing are in fact hot, expansive, and power hungry (Taylor, 2018). Digital backup belches carbon. As a result, a home or office computer already backed up to a local hard disk to mitigate everyday computer failures, and subsequently copied to the cloud to secure it from less ordinary catastrophes like flood or wildfire, contributes to the likely intensification of those same physical threats to local computing capacity (Brennan, 2016). Data centers provide apparent user efficiency – delivering access to data or computer processing on demand – through staggering inefficiencies in the form of excess capacity and infrastructural redundancies. As Hu (2017) observes, infrastructures like the cloud are built with their imagined potential failure in mind. In the case of data centers, the over-architecture that would make them ‘sustainable’ in a crisis (or even just a period of intense demand) contributes to their everyday unsustainability (Hu, 2017; see also Hu, 2015).

Many of the political and social issues identified with respect to paper and digital copies also apply to seeds: fears of (bio)piracy, data management woes and overconfidence in technological systems. The effect of duplication on environmental sustainability is less immediately visible but nonetheless potent. Concerns about the more apparent costs of unknown duplication in seed storage facilities drive a perverse situation – perverse, at least, with respect to the stated goal of keeping biological diversity extant – in which the successful reproduction and dispersal of that diversity is seen as a threat to its ultimate survival. In this light, seed bank backups are a strange conservation measure indeed. What is more, the iterative quality of seed and gene bank collection copying from the 1970s onward – making backups of backups – indicates that making and safeguarding copies has produced only an elusive security. It has historically offered reassurance for a time, but this palliative effect has typically receded as further threats have appeared on the horizon.

When the inevitable call arrives for a backup of the Svalbard Global Seed Vault, as a result of (say) the hazards of a melting permafrost, perhaps we can, and should, respond by demanding a new guiding metaphor for seed conservation and the practices to accompany it. There are already several options, from seed library (Soleri, 2018) to vegetable sanctuary (Curry, 2019). Alternatively, we might want to re-invest in the metaphor and practice of banking. Not the impersonal, unregulated, cloud-based banking of today, but banking as it was idealized the moment the modern seed bank was born. We might ask for a brick-and-mortar institution, situated in and serving the community, supported and overseen by a larger state infrastructure, not just taking in deposits for the long-term, but continually re-deploying these as loans – with the understanding that the circulation of valuable materials and mutual trust and dependency can also provide security.

## Notes

1. On earlier collections and facilities that were linked to active breeding programs (and whose responsibilities therefore encompassed more than storage), see Lehmann (1981), Flitner (2003), Pistorius and van Wijk (1999), and Saraiva (2013).

2. Banking metaphors have a longer history in genetics; see Bonneuil (2019) for a brief discussion. For a helpful discussion on the role of the banking metaphor in institutions that store and circulate bodily materials, see Swanson (2014).

3. A national seed storage – For safeguarding future crop production and quality, June 1954, US National Archives and Records Administration II (hereafter, NARA), RG 310, Crops Research Division, New Crops Research Branch, Plant Exploration Records, 1953–1972, Box 6, Folder National Seed Storage Laboratory – 1957.

4. Agricultural Appropriations for 1956, Hearings before the Subcommittee of the Committee on Appropriations, United States Senate, 84th Congress, First Session on HR 5239, 40.

5. Ibid., 32. Concern focused on seeds, although these are not the only source of plant germplasm. Clonally propagated crops (like potatoes) and orchard crops were also of interest to many breeders and to advocates of a more comprehensive conservation system.

6. Carl Erlanson to Dale K Vining, 8 May 1964, NARA, RG 310, Division of Plant Exploration and Introduction, Plant Exploration Records, Box 3, Folder Erlanson, Carl, 1945–65 [2].

7. Ibid. On the history of plant introduction in the United States, see Kloppenburg (2004: ch. 3).

8. Minutes of the Meeting of the Technical Committee, Southern Regional Cooperative Project S-9, New Plants, National Seed Storage Laboratory, Fort Collins, CO, 3–4 August 1959, https://www.ars.usda.gov/ARSUserFiles/60460500/Documents/minutes/S9TCM_1959.pdf

9. Seed storage facilities, 13 September 1949, NARA, RG 310, Crops Research Division, New Crops Research Branch, Plant Exploration Records, 1953-1972, Box 6, Folder National Seed Storage Laboratory – 1957; and, in the same file, ‘Report of the Sub-committee on National Seed Storage’, 27 February 1950.

10. US ‘bank’ at Fort Collins Houses makin’s [sic] of future crop varieties, date missing, clipping in National Laboratory for Genetic Resources Preservation, Fort Collins, CO, (hereafter, NLGRP), National Seed Storage Laboratory (NSSL) History Files (digitized), History 1960s, Articles.

11. DW Robertson, Report of the Standing Sub-Committee for a National Seed Storage Facility, [25–26 March 1955], NLGRP, NSSL History Files (digitized), History 1940s, Reports.

12. Frank A Kennedy, The word from Washington: Senate restores $450,000 seed bank appropriation, *Southern Seedsman*, July 1956, typescript copy in NLGRP, NSSL History Files (digitized), History 1940s, Reports.

13. See discussion about the ‘World collections of barley, oats, wheat and rice’ in Internal Audit Staff, Agricultural Research Service, Report No. 281: Survey of crops research division, Ft Collins and Greeley, Colorado, August 1959, NARA, RG 310, Crops Research Division, New Crops Research Branch, Plant Exploration Records, 1953-1972, Box 6, Folder National Seed Storage Laboratory – 1957. There were some exceptions. For example, just as the National Seed Storage Laboratory was getting ready to open its doors, a committee of US scientists asked if a copy of their ‘world collection’ of maize landraces could be included in the facility; see Curry (2017a).

14. First Meeting of the Consultative Group on International Agricultural Research, Summary of Proceedings, 19 May 1971, Annex III. Available at: http://hdl.handle.net/10947/260 (accessed 23 May 2022).
